# Remote ischemic preconditioning in the prevention of ischemic brain damage during intracranial aneurysm treatment (RIPAT): study protocol for a randomized controlled trial

**DOI:** 10.1186/s13063-015-1102-6

**Published:** 2015-12-29

**Authors:** Selma Tülü, Miriam Mulino, Daniel Pinggera, Markus Luger, Philipp Würtinger, Astrid Grams, Thomas Bodner, Ronny Beer, Raimund Helbok, Raffaella Matteucci-Gothe, Claudia Unterhofer, Elke Gizewski, Erich Schmutzhard, Claudius Thomé, Martin Ortler

**Affiliations:** Department of Neurosurgery, Medical University of Innsbruck, 35, Anichstrasse, Innsbruck, 6020 Austria; Department of Anesthesiology and Intensive Care Medicine, Medical University of Innsbruck, Innsbruck, 6020 Austria; Central Institute for Medical and Chemical Laboratory Diagnostics, Medical University of Innsbruck, Innsbruck, 6020 Austria; Department of Neuroradiology, Medical University of Innsbruck, Innsbruck, 6020 Austria; Department of Neurology, Medical University of Innsbruck, Innsbruck, 6020 Austria; Department of Public Health and Health Technology Assessment, UMIT Health and Life Sciences University, Hall in Tirol, Austria

**Keywords:** Biomarker monitoring, Cerebral ischemia, Intracranial aneurysm treatment, Ischemic preconditioning

## Abstract

**Background:**

The treatment of intracranial aneurysms may be associated with cerebral ischemia. We hypothesize that pre-interventional remote ischemic preconditioning (RIPC) reduces ischemic cerebral tissue damage in patients undergoing elective intracranial aneurysm treatment.

**Methods/Design:**

This study is a single-center, prospective, randomized, double-blind explorative trial. Patients with an unruptured intracranial aneurysm admitted to Innsbruck Medical University Hospital for coiling or clipping will be consecutively randomized to either the intervention group (= RIPC by inflating an upper extremity blood-pressure cuff for 3 x 5 min to 200 mmHg) or the control group after induction of anesthesia. Participants will be randomized 1:1 to either the preconditioning group or the sham group using a random allocation sequence and block randomization. The precalculated sample size is *n* = 24 per group. The primary endpoint is the area-under-the-curve concentration of serum biomarkers (S100B, NSE, GFAP, MMP9, MBP, and cellular microparticles) in the first five days after treatment. Secondary endpoints are the number and volume of new ischemic lesions in magnetic resonance imaging and clinical outcome evaluated with the National Institutes of Health Stroke Scale, the modified Rankin Scale, and neuropsychological tests at six and twelve months. All outcome variables will be determined by observers blinded to group allocation. This study was approved by the local institutional Ethics Committee (UN5164), version 3.0 of the study protocol, dated 20 October 2013.

**Discussion:**

This study uses the elective treatment of intracranial aneurysms as a paradigmatic situation to explore the neuroprotective effects of RIPC. If effects are demonstrable in this pilot trial, a larger, prospective phase III trial will be considered.

**Electronic supplementary material:**

The online version of this article (doi:10.1186/s13063-015-1102-6) contains supplementary material, which is available to authorized users.

## Background

Treatment of unruptured intracranial aneurysms involves maneuvers that may lead to cerebral ischemia in up to 60 % of patients [1, 2]. Although the majority of ischemic lesions remain asymptomatic, they may contribute to subtle cognitive deficits after elective aneurysm repair [2]. Ischemia-inducing events during aneurysm repair include brain tissue retraction, deliberate temporary cross-clipping or balloon occlusion of an afferent vessel for proximal control or coil placement, accidental clip occlusion of efferent vessels, thrombosis or thromboembolism during treatment, and other no-flow phenomena of unknown cause.

The effectiveness of interventions for protecting the brain during aneurysm treatment is controversial, and investigation of alternative techniques to increase the ischemic tolerance of the brain, not only during surgery, is desirable [3–5]. Such interventions should be safe, minimally invasive, controllable, cost-efficient, and, when administered during neurosurgical or neuroradiological interventions, practicable in the operating theater and the interventional suite.

*Preconditioning* is one such potential technique to achieve neuroprotection [6]. Preconditioning involves the application of a stimulus near but below the threshold of damage, aiming to protect an end organ from subsequent injury [7, 8]. A variety of stimuli has been shown to induce preconditioning [8]. In *direct* ischemic preconditioning (DIPC), subthreshold ischemia is applied directly to the perfusion territory that may later be exposed to more severe ischemia. In *remote* ischemic preconditioning (RIPC), subthreshold ischemia is induced in an organ or part of the body that is remote from the target organ at risk. The signal is thought to spread systemically by yet unidentified mechanisms [8–12].

Clinical trials using preconditioning prior to interventions that are associated with a high risk of intra-interventional ischemia have been performed in a variety of clinical disciplines [13-28].

Recently, the prevention of secondary damage associated with neural tissue injury itself has come into the focus of preconditioning strategies [29–36]. For example, Dupont-Hoougard and collaborators showed that RIPC, applied during transport to hospital, results in increased tissue survival after 1 month in patients undergoing thrombolysis for acute stroke when baseline levels of hypoperfusion where taken into account [32].

The occurrence of delayed cerebral ischemia (DCI) after subarachnoid hemorrhage (SAH) might be an ideal setting for studying the effects of IPC [37, 38]. Gonzalez and collaborators reported that patients undergoing RIPC showed reduced middle cerebral artery mean velocities, a reduced lactate/pyruvate ratio, and reduced glycerol levels. These effects lasted up to 2 days [39]. A preexisting cerebrovascular steno-occlusive disease or a preexisting infarction may also serve as a preconditioning stimulus that confers protection from radiologic vasospasm after a subsequent subarachnoid hemorrhage [40].

Techniques for ischemic preconditioning in association with intracranial aneurysm treatment evaluated the direct preconditioning effect of a two-minute vessel occlusion on PtO_2_, PtCO_2_ and pHt in the brain tissue of patients undergoing aneurysm clipping after aneurysmal SAH. The decline in PtO_2_ and pHt was significantly slower in the preconditioned group [41].

Two Cochrane reviews and at least one meta-analysis of IPC are available. Gurusamy analyzed IPC in liver transplantation. No evidence to support or refuse RIPC in donor liver retrievals was observed for clinically important markers (mortality, initial poor function, re-transplantation, and primary graft non-function). Aspartate transaminase levels as a biochemical marker of liver injury were different only on the third postoperative day [42]. Similarly, a Cochrane review of the effectiveness of IPC in vascular and endovascular surgery revealed no statistically significant difference between the two groups for any outcome parameter (including mortality) except reduced risk of myocardial infarction in the remote ischemic preconditioning group (which was significant according to the fixed-effect model) [43]. In a meta-analysis of 11 trials enrolling 1700+ patients undergoing elective cardiac intervention for coronary artery disease, RIPC significantly reduced the perioperative incidence of myocardial infarction and the incidence of contrast-induced acute kidney disease [44].

In summary, there is evidence from clinical trials that preconditioning may work in humans. Unanswered questions include type, timing and intensity of stimulus and, concerning preconditioning for the prevention of cerebral ischemia, outcome variables that have adequate sensitivity and specificity and are practicable in the clinical setting [37–39, 45, 46].

### How can effects of ischemic preconditioning on cerebral ischemia be detected?

1. Serum biomarkers
  Although proven serum biomarkers are available for ischemia-related damage to cardiac, hepatic or renal tissue [13, 17], clinical trials involving cerebral or spinal ischemic preconditioning are hampered by the lack of reliable and specific biomarkers for monitoring neuroprotective effects [46, 47].
  In stroke research, several biomarkers have been investigated [48–50] and may therefore serve as surrogate outcome variables in preconditioning studies. Calcium-binding protein S100 beta (S100B) is a glial protein that belongs to a family of calcium-mediated proteins named after their solubility in ammonium sulfate [49]. It can be found in astroglia, Schwann cells and in extraneural sources as in melanocytes, adipocytes and chondrocytes [49]. Neuron-specific enolase (NSE) is a glycolytic enzyme found mainly in the cytoplasm of neurons and cells of neuroendocrine origin, and in smaller concentrations in erythrocytes and platelets [51]. NSE levels correlated with stroke size in the majority of studies and high NSE levels generally indicated more severe stroke, but data are controversial.[51] S100B and NSE correlate with cerebral ischemia lesion volume in computed tomography (CT) and/or MR imaging [52, 53].
  Glial fibrillary acidic protein (GFAP) is the principal intermediate filament in mature astrocytes [54]. In a small study investigating biomarker detection of spinal cord damage during descending aorta aneurysm repair, the GFAP levels but not the S100B levels were associated with the occurrence of neuronal damage [55]. Levels of serum GFAP are higher in patients who experience secondary ischemia after subarachnoid hemorrhage [54]. GFAP levels and S100B levels (but not NSE levels) after subarachnoid hemorrhage correlated with the clinical condition and the degree of hemorrhage of patients on admission [56].
  Myelin basic protein (MBP) is a major constituent of central nervous myelin synthesized by oligodendrocytes [49]. Whiteley and collaborators reviewed 21 studies and found S100B, NSE and MBP to be the only markers tested having a specificity of > 90 % for neuronal damage [48].
  Metalloproteinases (MMPs) are secreted enzymes that cleave protein substrates [57]. Excreted MMPs are divided into several classes according to their substrate. MMP9, a gelatinase first described in neutrophils, plays a major role in the degradation of basal membrane proteins and tight junctions [57]. According to a review of 22 studies, increased serum levels of MMP9 were significantly correlated with infarct volume, stroke severity and worse functional outcome in acute stroke patients [58]. An MMP9 peak in serum is predictive for delayed cerebral vasospasm (dCVS) days before the onset of Doppler velocity changes or neurological deterioration [59]. MMP9 was observed to be significantly elevated in patients with aneurysmal subarachnoid hemorrhage (aSAH) who developed dCVS, as compared to SAH patients without dCVS [60].
  Microparticles (MPs) are blood-borne small membrane fragments shed from apoptotic or otherwise stimulated cells. They contain membrane components and intracellular components that are involved in cell signaling [61]. Elevated microparticle levels are found in a variety of thromboembolic diseases including acute coronary syndrome, myocardial infarction, diabetes mellitus, venous thromboembolism, atrial fibrillation, and ischemic stroke [61–64]. Endothelial microparticles correlate with clinical stroke severity and infarct volume [65]. Data in patients after SAH are controversial. In one study, the levels of endothelial microparticles were higher in patients experiencing vasospasm after SAH [66]. In another study, levels of microparticle subtypes associated with thrombosis and endothelial dysfunction were lower in patients with infarcts after SAH [67]. In RIPC, microparticles may play a special role as biological messengers. In an animal model, Giricz and collaborators were recently able to demonstrate that protection of the heart by RIPC is mediated by extracellular vesicles [68].
  In summary, none of the investigated biomarkers including those mentioned above, possess the specificity and sensitivity of biomarkers available to study, for example, cardiac ischemia. At present, use of a panel of biomarkers, determination of their concentration curve over time after an ischemic event and storage of serum probes for future determination of hitherto unknown biomarkers appear to be the most appropriate strategies in an exploratory study of central nervous IPC [46, 69].
2. Neuroimaging
  Diffusion-weighted imaging (DWI) and fluid-attenuated inversion recovery (FLAIR) are able to demonstrate new ischemic lesions with high sensitivity and specificity [70–72]. Multimodal MRI is included in standard protocols for stroke imaging [73]. The technique for comparing pre-interventional MRI to post-interventional MRI for the number and volume of new lesions in diffusion-weighted MRI and fluid-attenuated inversion recovery imaging was used successfully in a recent neuroprotective trial involving patients undergoing endovascular aneurysm repair [1]. Therefore, this technique is considered adequate for studying the effects of neuro-intervention and alterations due to IPC.
3. Clinical examination
  Clinical central nervous effects of IPC can be masked by confounding factors. Nevertheless, clinical effects have been demonstrated in patients suffering a stroke after preceding TIA [33] and in a recent large trial involving stroke patients undergoing RIPC during the prehospital phase. Although this was not the primary nor the secondary outcome in this study, patients in the treatment group presented with a lower NIHSS on admission [32]. Since improvement of the clinical outcome will remain the ultimate goal of all procedures aimed at preventing ischemic brain damage, clinical outcome variables should be investigated in a trial involving IPC.

### Why investigate RIPC in patients with an unruptured intracranial aneurysm?

Treatment of unruptured intracranial aneurysms under general anesthesia is a highly standardized clinical situation. In this situation, (1) the incidence of clinically silent ischemia is high (as high as 60 % in patients undergoing endovascular procedures [1, 74]), (2) no previous ischemic damage has occurred (and a baseline for biomarker determination can be established beforehand), and (3) the preconditioning stimulus can be administered at a pre-specified moment prior to occurrence of the real damage. Aneurysm treatment may therefore present some essential features needed to study the induction of ischemic tolerance in the brain under clinical conditions despite all problems associated with the determination of valid study endpoints [37, 45, 75, 76].

### Why include two different procedures in the study protocol?

We included both procedures - microsurgical clipping and endovascular coil occlusion of intracranial aneurysms - because both may induce focal cerebral ischemia (ischemia to one defined cerebral vascular territory) and because both may lead to local endothelial damage. Both procedures carry a comparable risk to induce focal cerebral ischemia.

The difference is that, in endovascular procedures, the source of ischemia is inside the vessel and in direct contact with the endothelium and the bloodstream, whereas in microsurgical clipping, the ischemia-inducing agent is outside the vessel lumen. This might be a drawback in the study design but could also increase the external validity of this study. Other possible technical confounders in a study of this type that defeat standardization include but are not limited to (1) a different duration of vessel occlusion between the study individuals, (2) a lack of possibility to differentiate between complete and incomplete vessel occlusion, (3) the impossibility to control for collateralization of the occluded vascular territory, and (4) the fact that some patients will undergo repetitive occlusion periods and others a singular occlusive period as dictated by clinical necessity.

The goal of the RIPAT study is to investigate whether remote ischemic preconditioning during the elective treatment of intracranial aneurysms reduces the occurrence of ischemic damage as measured by serum biomarker determination, neuroimaging, and clinical, as well as neuropsychological, examination.

## Methods/design

### Study setting

The trial will be conducted at Innsbruck Medical University Hospital, an academic tertiary care center that also serves as the referral center for all patients with intracranial vascular pathologies requiring neuro-intervention in the western part of Austria.

### Trial design

RIPAT is a randomized, prospective, controlled, double-blind, exploratory clinical trial.

### Patients

All patients admitted to the Departments of Neurosurgery or Neurology with one or more unruptured intracranial aneurysm(s) will be discussed by an interdisciplinary vascular board consisting of neurosurgeons, neuroradiologists, and neurologists and considered for study inclusion if aneurysm treatment is indicated by board decision.

Patients with asymptomatic intracranial aneurysm(s) are eligible for the study if they (1) are aged 18 or older, (2) agree to undergo endovascular coiling or surgical clipping, and (3) give their consent to the study. Exclusion criteria are as follows: (1) presence of clinical or radiological signs of subarachnoid hemorrhage; (2) mycotic or dissecting morphology of the aneurysm(s); (3) preplanned vessel sacrifice as the aneurysm treatment modality of choice; (4) history of stroke or TIA within the last 6 months; (5) signs or symptoms of peripheral vascular disease; (6) previous serious cerebral diseases that would preclude protocol completion or MRI analysis of minor strokes; (7) any contraindications against MRI scan; (8) language barriers that would prevent completion of the neuropsychological tests; (9) drugs, lifestyle factors, and systemic illness that interfere with biomarker determination and (10) pregnancy.

### Procedures

**Fig. 1:**
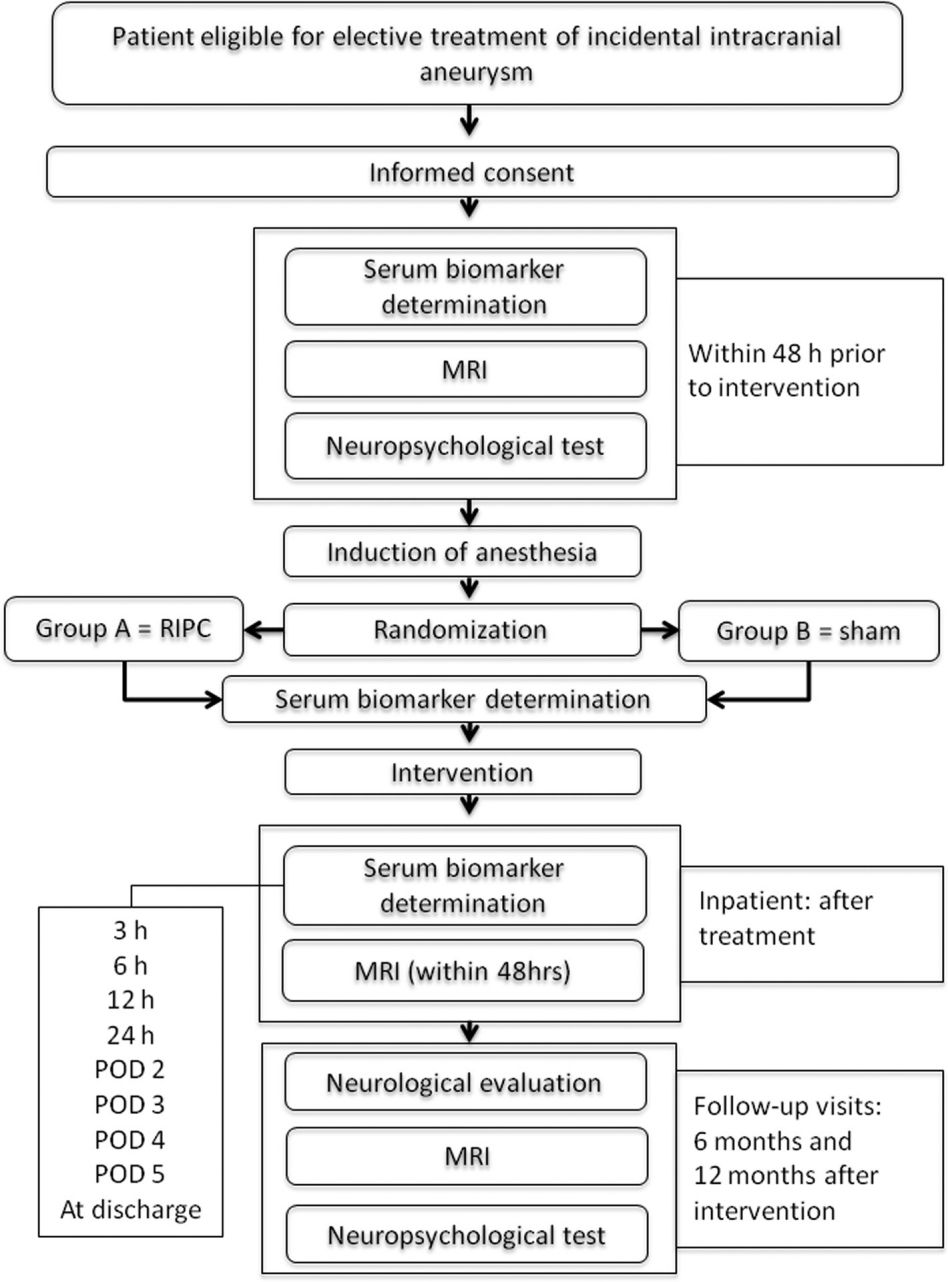
Flowchart of the trial (MRI = magnetic resonance imaging)

Figure 1 depicts the structure of the trial.

#### Remote ischemic preconditioning (RIPC) and sham preconditioning (SPC)

Study patients will be randomly assigned to either the preconditioning group (Group A, treatment group) or the control group (Group B, sham preconditioning) on opening a sealed envelope containing the group assignation after anesthesia is induced in the induction room. In both RIPC and SPC, a standard blood pressure cuff is fixed on the patient’s upper arm. Care will be taken to ensure that the blood-pressure cuff is adequate to accommodate the patient’s arm [77]. In Group A, RIPC consists of cuff inflation to 200 mmHg for 3 x 5 min alternating with 5 min of intermittent reperfusion by complete cuff deflation. In Group B, maximum cuff inflation pressure is limited to 10 mmHg for 3 x 5 min alternating with 5 min of complete cuff deflation.

RIPC and SPC will be performed by the anesthesiologist aided by a second, unblinded member of the study team after the induction of general anesthesia and prior to definitive patient positioning for the intervention. These two members of the team are not involved in the intra-interventional decisions concerning deliberate ischemia and are blinded for outcome analysis. Standard operation procedures (SOP) describing the preconditioning procedure are available to all investigators (see Additional file 1: Supplementary material). A written checklist must be completed by those performing the preconditioning to ensure protocol adherence (see Additional file 1: Supplementary material).

Adverse events or unintended effects of the trial intervention will be noted in the preconditioning protocol and communicated to those performing the subsequent aneurysm treatment. The decision to abort the aneurysm treatment procedure in such a situation will be made by the surgeon or the endovascular therapist after conferring with the principal investigator. Patients with a complication will be managed in a manner appropriate to the situation and informed thereof after recovery from anesthesia.

#### Aneurysm treatment

Treatment of intracranial aneurysms will consist of either microsurgical clipping or endovascular coiling under general anesthesia following standard methods. All events that could possibly be associated with ischemia, including a drop or deliberate reduction in the systemic blood pressure of more than 20 % of the prior systolic blood pressure lasting more than 3 min and every deliberate or unintentional vessel occlusion during the procedure will be recorded by the anesthesiologist. All injuries and unintended effects associated with the preconditioning procedure or the aneurysm treatment will be registered.

The anesthetic protocol is not prespecified. We expect that the majority of patients will receive total intravenous anesthesia (TIVA) that includes propofol because this is the standard anesthesiologic regimen in our neurosurgical patients. However, the anesthesiologist is free to use the protocol that he/she thinks is the most appropriate. Different interventions will not require different anesthetic protocols.

#### Biomarker analysis

Blood samples for biomarker analysis will be drawn by the nursing staff at pre-specified time points as described below. The first sample will be taken within 24 h preceding intervention, providing an individual baseline for each patient. Further blood samples will be taken immediately prior to the preconditioning procedures, after the preconditioning and prior to the start of the aneurysm treatment, as well as at 0, 3, 6 and 12 h and daily thereafter for 5 days after completion of aneurysm treatment. Serum biomarkers will be determined at the Central Institute for Medical and Chemical Laboratory Diagnostics of the Medical University of Innsbruck. NSE will be measured by means of an electrochemical luminescence immunoassay based on monoclonal antibodies, detecting the γ subunit of NSE (Elecsys, Roche Diagnostics GmbH, Mannheim, Germany). S100B will be determined by an electrochemical luminescence immunoassay, using monoclonal antibodies specifically targeted on the astrocyte-specific β-chain of the S100 dimer (Elecsys, Roche Diagnostics GmbH, Mannheim, Germany). MMP9 will be quantified by an MMP9 sandwich enzyme immunoassay (R&D Systems, Minneapolis, MN, USA) that detects the 92 kDa proactive and the 82 kDa active forms, but not the 65 kDa form with monoclonal antibodies. GFAP and MBP will be measured by sandwich enzyme immunoassays based on polyclonal antibodies (GFAP: biovendor, Heidelberg, Germany; MBP: USCN Life Science, Wuhan, China). Cellular microparticles will be analyzed using fluorescence-activated cell sorting (FACS) from plasma in the Research Laboratory of the Department of Neurology using the methodology described previously by Lackner [66]. Serum samples will be stored at –80 °C for future analysis in ancillary studies at the Central Institute for Medical and Chemical Laboratory Diagnostics.

#### Neuroimaging

All patients undergo a baseline MRI scan within 48 h preceding aneurysm treatment. In all patients, a post-interventional MRI scan will be performed 12 to 48 h after termination of the aneurysm treatment. All imaging will be done in the Department of Neuroradiology of the Medical University of Innsbruck. Study imaging protocol includes an axial FLAIR sequence with 3-mm slice thickness, an axial DWI sequence with 2-mm slice thickness and an axial T1-weighted 3 days magnetization-prepared rapid gradient echo (MPRAGE) sequence with 1-mm slice thickness. Pre-interventional and post-interventional MR sequences will be compared. The presence and age of ischemic lesions in the pre-interventional MRI will be identified, apparent diffusion coefficient (ADC) and fractional anisotropy (FA) values will be measured with region of interest (ROI) technique in different brain regions of both hemispheres (frontal, parietal, temporal occipital white matter, putamen, internal capsule, and pons) and brain volume will be determined by voxel-based morphometry. In the post-interventional images, new ischemic lesions will additionally be detected. Number (in absolute numbers) and volume (in mm^3^) of the new ischemic lesions will be calculated, and the ADC/FA values within the lesions will be measured. Imaging data will be independently assessed by two experienced consultant neuroradiologists blinded to the intervention. Discrepancies in image interpretation will be solved by consensus. All values will be examined for normality and appropriate statistical methods will be used. Average time-to-post-interventional MRI for both groups will be reported.

#### Follow-up

Follow-up examinations are scheduled at 6 and 12 months after aneurysm treatment and include clinical assessment, MRI scan, and neuropsychological examination. Although not specifically excluded from the study, patients who seem unable to comply with follow-up will be included only after being interviewed about their willingness to participate also in the follow-up examinations.

#### Clinical evaluation

Clinical outcome after 6 and 12 months will be assessed by a neurologist blinded to the preconditioning procedure using the National Institutes of Health Stroke Scale (NIHSS) (http://www.ninds.nih.gov/doctors/stroke_scale_training.htm, accessed 8 January 2015) and the modified Rankin Scale (mRS) [77]. Clinically apparent deficits or unintended effects will be noted. The examination will take place in the outpatient clinics of the Department of Neurosurgery or Neurology.

#### MRI

See above neuroimaging.

#### Neuropsychological examination

All patients undergo standardized neuropsychological examination in the week before treatment and at the 6- and 12-month follow-ups in the rooms of the neuropsychological laboratory of the Department of Neurology. Testing will be performed by an experienced neuropsychologist blinded to the preconditioning procedure. The test battery includes the Verbal Learning Memory Test, [79] the Digit Span Test from the Wechsler Memory Scale (WMS), [80] Trial Making Test A/B, [81] Regensburg Word Fluency Test, [82] the Go/No-go Task 2 stimuli 1 target from the Test of Attentional Performance (TAP) [83] and the Hospital Anxiety and Depression Scale (HADS-D) [84].

### Statistics

#### Blinding

Study patients, as well as all members of the medical, nursing and scientific staff involved in study patient treatment, will remain blinded to patient group allocation. All data collected for the trial will be collected by members of the research team who do not know the treatment allocation. The anesthesiologists, including ML and two neurosurgeons (MM and DP), are responsible for execution of the RIPC procedure and therefore will be the only unblinded study members and will not be involved in the study patient treatment, outcome analysis or data analysis.

RIPC takes place in an ancillary room of the operating theater or the neurointerventional suite, separated from the main rooms by soundproof doors fitted with small windows. The patient is therefore shielded almost completely from occasional onlookers. The interventional team (surgeons or endovascular therapists) is summoned to enter this room and proceed with patient positioning for intervention after completion of the preconditioning procedure.

#### Randomization

The Center for Statistical Consulting and Continuing Education (CSCCE) at UMIT (University for Health Sciences, Medical Informatics and Technology) generated the random allocation sequence using the PLAN procedure in the SAS statistical package V.9.1. Participants were randomized 1:1 to either the preconditioning group or the sham group using block randomization with a block size of four at a time and a random-block sequence. Assignment of the study subjects was documented in a randomization schedule. Sequentially numbered sealed opaque envelopes containing group allocation for each patient were prepared using the random allocation sequence by a statistician not involved in patient treatment. Envelopes will be opened by one of the unblinded members of the study group (see above) after induction of anesthesia.

#### Statistical analysis

Statistical analysis will be performed by an independent external statistician (RMG). This study is designed as an explorative study to provide data for future confirmatory studies. The main outcome variable (area under the curve, AUC) for each biomarker will be analyzed using intra-operative ischemia as the only explanatory variable in the two groups. No corrections will be made for multiplicity. A difference of ≥ 2 standard deviations (SD) in the main outcome variable is considered to be a statistically relevant effect. The alpha level is set at 0.05 %. The 95 % confidence intervals for the differences will be calculated.

No a priori empirical evidence suggests which of the six chosen biomarkers would be the ideal single primary outcome, but to define the sample size, we allocated the AUC of S100 as the primary outcome. Power analysis aiming to detect a difference of more than two SD in the primary outcome between the groups would require a sample size of *n* = 16σ2/d2 for a two-sided α = 0.05 and 1-β = 0.8 [85]. *N* = 4 patients per group would permit a difference of ≥ 2 SD in serum biomarker levels to be detected in the study population. Assuming that only one in five patients will sustain cerebral ischemia during aneurysm treatment, this would require a sample of *n* = 20 patients per group. Additional 20 % (*n* = 4 patients) per group will be included for unexpected dropouts, unexpected low rates of intra-interventional ischemia or unexpected loss to follow-up. This would require a total study population of *n* = 48 patients (*n* = 24 per group).

All statistical analysis will be performed on an intention-to-treat basis and per protocol. Standard methods of exploratory data analysis will be used for group comparison. Quantitative parameters will be tested for normality and compared using the *t* test or compared with the Mann–Whitney *U* test for non-normally distributed data. Qualitative parameters will be compared using the chi-squared test or Fisher’s test. Group differences for the main outcome variable will be compared using the two-sided *t* test for independent groups or the Mann–Whitney *U* test, as appropriate. Secondary outcome parameters will be compared using the Mann–Whitney *U* test for quantitative data and the chi-squared or Fisher's test for dichotomized outcome data.

Preplanned subgroup analyses include an analysis stratified by aneurysm treatment technique (microsurgical or endovascular) and by anesthetic regimen (with or without propofol).

Missing data for biomarker serum values (for example, due to hemolytic probes) will be handled using a statistical model and the maximum likelihood method, in which estimates and standard errors are based on the likelihood function given in the observed data. Missing imaging data (for example, inability to investigate by MRI during the first 2 postoperative days) will be replaced by calculating the number and volume of areas assumed to be due to ischemia on the basis of CT scans (if available)

The SAS software package V9.1 will be used for statistical analysis.

### Ethical considerations

The study complies with the Helsinki Declaration [86] and was approved by the local institutional Ethics Committee of the Medical University of Innsbruck (Ethikkommission der Medizinischen Universität Innsbruck, Geschäftsstelle, Innrain 43, 1.Stock, A-6020 Innsbruck) (UN5164) in Version 3.0 of the study protocol, dated October 20 2013. An amendment of the study protocol was approved by the same institution on April 24, 2015 (UN 5164, 327/4.18, 348/5.7). The present paper includes all changes contained in this amendment. Future amendments, if necessary, will be available through the trial registration database (see below).

Informed consent from potential trial participants will be obtained either by the principal investigator (MO) or by the collaborators CU, RB, RH and ES after the board’s recommendation for aneurysm treatment. The model consent form V.3.1 dated October 28, 2013 is available under Additional file 1: Supplementary materials.

Patients may discontinue their participation in the study at any time upon request.

The study is registered with www.clinicaltrials.gov (registration ID: NCT02162654) and with the Clinical Trial Center of the Medical University of Innsbruck (http://ctc.tirol-kliniken.at/page.cfm?vpath=oeffentliche&action=viewdetail&studie=5430) (registration ID: 20131101–823).

#### Safety of RIPC

With RIPC, the intentional exposure of ischemia-sensitive organs - such as the brain - is avoided by applying stimuli to more ischemia-tolerant tissues like skeletal muscle. Obviously, direct vessel manipulation for introduction of the preconditioning stimulus is also avoided. Although negative effects of IPC cannot be fully excluded, up to now neither experimental nor translational data have documented any negative effects. The IPC protocol that will be employed in our trial has been used in other clinical trials without known adverse events. A phase Ib trial in awake patients investigating the feasibility and safety of IPC using RIPC in the leg did not demonstrate any adverse events associated with RIPC [87]. A discomfort questionnaire was completed by 80 study patients undergoing preclinical RIPC in the arm for suspected cerebral ischemia. None reported any significant discomfort [32].

However, repetitive ischemia in a limb of patients with signs of occlusive peripheral vascular disease may cause additional damage to this limb. Therefore, these patients are excluded from study participation (see exclusion criteria).

Subtle changes in coagulation time (PT and INR, not PTT) were observed in patients undergoing at least four cycles of RIPC after SAH. No hemorrhagic complications were observed in this study [88]. The study was published after enrollment of our first patients. A debate ensued in the steering committee concerning whether patients undergoing stent-assisted coiling (and therefore requiring acetylsalicylic acid and clopidogrel preloading) would be exposed to an increased risk for hemorrhagic complications. One potential study patient was not included for these reasons. After thorough discussion, we concluded from the available evidence that our RIPC scheme (three cycles of upper arm ischemia with the patient under general anesthesia) does not raise safety concerns for the subsequent aneurysm treatment procedure (including stent-assisted coiling).

#### Other potential risks associated with participation in this study

Study inclusion requires anesthesia to be prolonged for approximately 30 min. During this time, the patient is fully monitored and under the supervision of a senior anesthesiologist.

Biomarker determinations will not interfere with the clinical routine in any way. Patients undergoing aneurysm treatment are assumed to undergo preoperative and daily blood sampling for routine monitoring purposes thereafter for at least 5 postoperative days at regular intervals and as deemed necessary thereafter. Participation in the study requires the additional removal of a small amount of blood at predetermined intervals without separate venipuncture. All patients have central venous lines in place according to standard operating procedures. Blood removal is not considered a risk. A separate venipuncture, if necessary in an exceptional situation, can cause discomfort if the patient is awake.

All patients subjected to aneurysm treatment undergo imaging prior to treatment and after the procedure. Study participation requires an MRI in all patients prior to treatment, within 48 h after treatment, at 6 months and at 1 year. Thus, patients who normally would not undergo MRI examination at these time points, that is, in whom ischemia is not suspected on clinical or imaging (CT) grounds, will also undergo MRI. MRI does not involve radiation. Patients not appropriate for MRI investigation will not be included in the study (see exclusion criteria). In artificially ventilated patients, MRI examination will be supervised by an experienced anesthesiologist. The Clinical Departments of Neuroradiology and Anesthesiology of the Medical University of Innsbruck have long-standing experience with MRI examinations in severely ill or intubated neurological patients. Examination of intubated patients is possible 24/7.

Neuropsychological testing will be performed prior to the intervention and at 6 and 12 months during the regular outpatient visits. This additional testing will prolong these visits for approximately 2 hours.

All patients in our departments undergo regular follow-up visits at 4 weeks, 6 months and 1 year after treatment of an intracranial aneurysm. Data concerning functional outcome will therefore be recorded as part of the routine clinical documentation. No additional visits to the outpatient department for study purposes will be necessary. Study participants not willing or able to present for the follow-up visit, for whatever reason, will be contacted by telephone and interviewed using the structured questionnaire published by Bruno [89].

#### Adverse events

Events considered adverse events associated with RIPC therefore include intra-interventional or post-interventional changes in coagulation parameters not commonly seen during neurosurgical or neurointerventional procedures. Severe adverse events include (1) all events that are rarely observed during routine treatment of intracranial aneurysms and that cause a new neurological deficit or require re-intervention, (2) all hemorrhagic complications causing a new neurological deficit, and (3) every ischemic damage to a limb that underwent RIPC.

#### Data handling and data safety

A patient identification list with information about all study patients will be authored by the investigators. This list will contain every patient’s full name, ID number, date of birth, contact address, name and contact information of the family doctor, date of inclusion in the study, date of termination of study participation and (if applicable) date and reason for preliminary exclusion from the study. A patient allocation list showing the patients’ group assignment will be administered by the Clinical Department of Anesthesiology (ML).

All data will be pseudonymized as soon as clinically reasonable. Data entry in an electronic database (SPSS spreadsheet) will be performed in pseudonymized form by ST and checked by a second operator. Data will be stored in a database hosted by the IT Services of Innsbruck Medical University Hospital that is accessible only within the Clinical Department of Neurosurgery. Backup storage of this database is performed automatically every 24 h. Data analysis and dissemination will be performed using anonymous data exclusively.

Confidentiality of patient data is assured by restricted access to the storage device granted to authorized investigators only. Data entry will be performed, dated and signed by authorized investigators only. Authorized investigators are identified in a signature log, which will be stored in the Investigator’s Site File. Case Report Forms (CRF) and coding information will be stored by the principle investigator after data input.

#### Methods for ensuring quality control and adherence to the study plan

The Department of Neurosurgery has installed a Data Monitoring Committee that meets every week to discuss ongoing studies. All patients who are potential candidates for study inclusion and all RIPAT study participants will be reviewed in this meeting. Additionally, all RIPAT investigators meet at least twice a year to discuss pertinent study progress issues. Prior to study initiation nursing staff was instructed about goals and details of the study. Written SOPs that accompany the various steps of the study (patient screening, preconditioning procedure, taking of blood samples) were issued and made available to all staff.

#### Dissemination

The study protocol following SPIRIT guidelines [90, 91] will be published in a peer-reviewed journal with open access. A report drafted by the principal investigator and authored by all investigators after completion of the study will be submitted for peer-reviewed publication in two parts. The first part, which is due 3 months after the end of patient recruitment, will report on the effects of RIPC on the primary outcome (biomarker concentration) and on short-term neuroimaging changes. A second report concerning clinical outcome will be filed after follow-up of the entire study cohort has been completed. All reports will be linked by citing the registration details. The reports will be prepared according to CONSORT guidelines and its extension to non-pharmaceutical interventions" [92] and comply with the ICH GCP Guidelines for Structure and Content of Clinical Study Reports [93]. The principal investigator will have unrestricted access to the final dataset.

## Discussion

This study uses the elective treatment of intracranial aneurysms as a paradigmatic situation to explore the neuroprotective effects of remote ischemic preconditioning. In this setting, ischemia as visualized by neuroimaging does occur, but is usually not clinically relevant. Nevertheless, serum biomarkers and/or advanced MRI techniques may be able to detect significant group differences between patients who underwent remote ischemic preconditioning and those who did not. In this case, any of these biomarkers may be a candidate as a secondary outcome parameter for future exploration in adequately powered phase III trials aimed at demonstrating a clinically relevant difference as the primary outcome. The primary outcome in such a trial could be the rate of cerebral events assessed within 30 days after randomization. These events comprise death or any new neurological deficit (whether temporary or permanent) attributable to an ischemic lesion detected by neuroimaging. We estimate that, in the setting of a multicenter study involving different institutions and different interventionalists, the rate of such events is 5 % in this patient population and that a reduction of this rate by 50 % would be clinically worthwhile.

In case any primary outcome parameter of the RIPAT trial does not show a statistically significant difference between the treatment and intervention group, the planning of future trials using ischemic preconditioning in this patient group will be based on the analysis of full results including all clinical, imaging, and neuropsychological data.

The study has limitations. First, this is a single-center trial. Second, serum biomarkers in the field of cerebral ischemia may lack the sensitivity and specificity of biomarkers used to monitor cardiac, renal or hepatic ischemia. Third, power analysis for the study is based on the assumption that the determination of a continuous variable, namely the area under the curve of a biomarker, is able to show differences between the treatment and control group. Effects detected by serum biomarkers might be so small that this exploratory study will be found to be underpowered. Fourth, although we use the upper extremity and an established protocol to induce remote ischemic preconditioning, the ideal place and timing for providing protective effects is actually unknown. Fifth, we appreciate that a surgical intervention under total intravenous anesthesia that uses propofol is associated with a large number of stimuli that may also have neuro-altering effects. This may lead to bias. Sixth, two procedures are used to induce focal cerebral ischemia, and although both are believed to be associated with a comparable risk to induce focal cerebral ischemia, the risks may be comparable but not identical.

The strengths of this study are, first, that it explores ischemic preconditioning in a highly standardized clinical situation, namely the elective treatment of an unruptured intracranial aneurysm. In this situation, preconditioning stimulus and ischemic stimulus are time-locked in a highly standardized fashion. Second, we employ a simple, clinically applicable preconditioning technique that was used successfully in other clinical trials investigating end-organ ischemia. If this technique is effective, it may be applicable in many other areas of medicine. Third, the serum biomarkers used to determine the primary outcome are biomarkers that are widely available and that have been used successfully in other clinical trials of cerebral ischemia research.

The results of this trial will not provide a definitive answer to the question of whether remote preconditioning is useful for protecting the brain from ischemia, but are expected to provide data essential to designing larger multicenter phase III trials.

## Trial status

The first patient was enrolled in November 2013. At the time of manuscript submission, enrollment of participants continues.

## Additional file

Additional file 1: **Supplementary material.** (DOC 123 kb)

## Abbreviations

ADC: apparent diffusion coefficient
AUC: area under the curve
(a) SAH: (aneurysmal) subarachnoid hemorrhage
CT: computed tomography
DCI: delayed cerebral ischemia
dCVD: delayed cerebral vasospasm
DIPC: direct ischemic preconditioning
DWI: diffusion-weighted imaging
EEG: electroencephalography
FA: fractional anisotropy
FACS: fluorescence-activated cell sorting
FLAIR: fluid attenuation inversion recovery
GFAP: glial fibrillary acidic protein
HADS-D: Hospital Anxiety and Depression Scale
IPC: ischemic preconditioning
MBP: myelin basic protein
MMP9: matrix metalloproteinase 9
MP: microparticles
MPRAGE: magnetization-prepared rapid gradient echo
MRI: magnetic resonance imaging
mRS: modified Rankin Scale
NIHSS: National Institutes of Health Stroke Scale
NSE: neuron-specific enolase
PVD: peripheral vascular disease
RIPC: remote ischemic preconditioning
ROI: region of interest
SD: standard deviation
SOP: standard operating procedure
SPC: sham preconditioning
S100B: calcium-binding protein S100beta
TAP: test of attentional performance
TIA: transient ischemic attack
WMS: Wechsler Memory Scale
